# Radial-based acquisition strategies for pre-procedural non-contrast cardiovascular magnetic resonance angiography of the pulmonary veins

**DOI:** 10.1186/s12968-020-00685-1

**Published:** 2020-11-30

**Authors:** Pascale Aouad, Ioannis Koktzoglou, Bastien Milani, Ali Serhal, Jose Nazari, Robert R. Edelman

**Affiliations:** 1 Radiology, Feinberg School of Medicine, Northwestern University, Chicago, IL USA; 2 Radiology, NorthShore University HealthSystem, Walgreen Building, G534, 2650 Ridge Avenue, Evanston, IL 60201 USA; 3 University of Chicago Pritzker School of Medicine, Chicago, IL USA; 4 Center for Biomedical Imaging, Lausanne, Switzerland; 5 Medicine, NorthShore University HealthSystem, Evanston, IL USA

**Keywords:** Pulmonary veins, Radial, Stack-of-stars, Breath-holding, Free-breathing

## Abstract

**Background:**

Computed tomography angiography (CTA) or contrast-enhanced (CE) cardiovascular magnetic resonance angiography (CMRA) is often obtained in patients with atrial fibrillation undergoing evaluation prior to pulmonary vein (PV) isolation. Drawbacks of CTA include radiation exposure and potential risks from iodinated contrast agent administration. Free-breathing 3D balanced steady-state free precession (bSSFP) Non-contrast CMRA is a potential imaging option, but vascular detail can be suboptimal due to ghost artifacts and blurring that tend to occur with a Cartesian k-space trajectory or, in some cases, inconsistent respiratory gating. We therefore explored the potential utility of both breath-holding and free-breathing non-contrast CMRA, using radial k-space trajectories that are known to be less sensitive to flow and motion artifacts than Cartesian.

**Main body:**

Free-breathing 3D Cartesian and radial stack-of-stars acquisitions were compared in 6 healthy subjects. In addition, 27 patients underwent CTA and non-contrast CMRA for PV mapping. Three radial CMR acquisition strategies were tested: (1) breath-hold (BH) 2D radial bSSFP (BH-2D); (2) breath-hold, multiple thin-slab 3D stack-of-stars bSSFP (BH-SOS); and (3) navigator-gated free-breathing (FB) 3D stack-of-star bSSFP using a spatially non-selective RF excitation (FB-NS-SOS). A non-rigid registration algorithm was used to compensate for variations in breath-hold depth.

In healthy subjects, image quality and vessel sharpness using a free-breathing 3D SOS acquisition was significantly better than free-breathing (FB) Cartesian 3D. In patients, diagnostic image quality was obtained using all three radial CMRA techniques, with BH-SOS and FB-NS-SOS outperforming BH-2D. There was overall good correlation for PV maximal diameter between BH-2D and CTA (ICC = 0.87/0.83 for the two readers), excellent correlation between BH-SOS and CTA (ICC = 0.90/0.91), and good to excellent correlation between FB-NS-SOS and CTA (ICC = 0.87/0.94). For PV area, there was overall good correlation between BH-2D and CTA (ICC = 0.79/0.83), good to excellent correlation between BH-SOS and CTA (ICC = 0.88/0.91) and excellent correlation between FB-NS-SOS and CTA (ICC = 0.90/0.95). CNR was significantly higher with BH-SOS (mean = 11.04) by comparison to BH-2D (mean = 6.02; P = 0.007) and FB-NS-SOS (mean = 5.29; P = 0.002).

**Conclusion:**

Our results suggest that a free-breathing stack-of-stars bSSFP technique is advantageous in providing accurate depiction of PV anatomy and ostial measurements without significant degradation from off-resonance artifacts, and with better image quality than Cartesian 3D. For patients in whom respiratory gating is unsuccessful, a breath-hold thin-slab stack-of-stars technique with retrospective motion correction may be a useful alternative.

## Background

Atrial fibrillation (AF) is a common arrhythmia that is associated with increased morbidity and mortality [1]. Radiofrequency catheter ablation of the pulmonary vein (PV) ostia represents a well-known treatment modality for AF that requires accurate visualization of the PV and detection of anatomical variants in order to obtain successful PV isolation and to prevent complications [2]. Computed tomography angiography (CTA) and contrast-enhanced (CE) cardiovascular magnetic resonance angiography (CE-CMRA) are currently well accepted modalities for pre-procedural PV imaging [3]. However, CTA involves potential risk from radiation exposure and administration of an iodinated contrast media. CE-CMRA requires appropriate timing of the image acquisition, patient cooperation with breath holding, and administration of a gadolinium-based contrast agent (GBCA) which is contraindicated in patients with stage 4 or 5 kidney disease [4]. Moreover, PV CE-CMRA is typically acquired without electrocardiographic (ECG) gating, resulting in blurring of the cardiac anatomy [5].

Free-breathing (FB) Cartesian 3D balanced steady-state free precession (bSSFP) has been described as a potential non-contrast imaging alternative to CE-CMRA for PV imaging [6, 7]. However, Cartesian techniques are known to be more prone to degradation by motion-induced ghost artifacts and blurring than radial techniques [8]. We therefore performed an initial comparison of FB Cartesian 3D bSSFP with FB stack-of-stars (SOS) 3D bSSFP in healthy subjects to determine if the radial-based approach offered significant advantages. Moreover, while FB techniques are generally reliable, in some patients irregular breathing patterns preclude adequate image quality or result in excessive scan times [9]. We therefore also implemented breath-hold (BH) radial-based 2D and 3D bSSFP techniques and compared them to FB SOS bSSFP for the evaluation of the PV in a cohort of patients scheduled for PV isolation, using CTA as the reference standard.

## Methods

This study was approved by our institutional review board and written informed consent was obtained from all participants. Six healthy subjects (4 males, 34–60 years) were imaged using non-contrast CMRA. In addition, 27 patients (16 males; 67.4 years, range 47–82) who had a history of AF underwent both CTA and non-contrast CMRA for PV imaging prior to PV isolation.

### Image acquisition

#### CMR techniques

All studies were performed on a 1.5 T CMR scanner (MAGNETOM Avanto, Siemens Healthineers, Erlangen, Germany). The subjects were placed in a supine position. ECG gating was used to acquire images during mid to late diastole. Images were obtained in the coronal plane with the scan region encompassing the left atrium and proximal through mid-segments of the PVs.

In order to determine whether navigator-gated FB 3D SOS bSSFP using a spatially non-selective radiofrequency (RF) excitation (FB-NS-SOS) offered any advantages for non-contrast PV imaging over the previously described approach of FB Cartesian 3D bSSFP (also using a spatially non-selective RF excitation) [6, 7], both sequences were acquired using similar scan parameters in the 6 healthy subjects. For patients, PV CMRA was obtained using a combination of BH and FB prototype 2D and 3D radial-based acquisitions with a bSSFP readout. Sequences included BH 2D radial bSSFP (BH-2D), BH 3D SOS bSSFP (BH-SOS) and FB-NS-SOS. From a total of 27 subjects, 7 were scanned only with BH-2D, 7 were scanned with BH-2D and BH-SOS while the remaining 13 were scanned with all three sequences BH-2D, BH-SOS and FB-NS-SOS.

Pulse sequence parameters were determined empirically from preliminary healthy subject studies. Typical pulse sequence parameters for all sequences are summarized in Table 1. All techniques used ECG gating with data acquired during diastole. No attempt was made to optimize the trigger delay for individual patients. The FB sequences were triggered to every 2nd R-wave, whereas the BH sequences were triggered to every R-wave. Respiratory gating for FB acquisitions was performed with standard cross-pair navigators using adaptive correction and a ± 3-mm navigator gating window. All radial sequences used chemical shift-selective fat suppression as well as a standard in-line reconstruction with basic regridding and without additional calibration scans for gradient delay correction. A standard Siemens “cardiac” shim algorithm was utilized for all sequences with a manually placed shim box encompassing the heart and proximal PV but excluding air above and below the chest and also excluding the abdomen and upper chest.

**Table 1:** Typical imaging parameters for BH-2D (breath hold 2D radial SSFP), BH-SOS (breath hold 3D stack-of-stars SSFP) and FB-NS-SOS (navigator gated free breathing non-selective 3D stack-of-stars SSFP). Scan time values with asterisk assume 100% navigator gating efficiency

	BH-2D	BH-SOS	FB-NS-SOS	FB-NS-Cartesian 3D
Scan time (s) for heart rate of 60 bpm	18	16	312*	312*
Acquisition window (ms)	327	327	118	118
Respiratory control	5 BH	9 to 11 BH	Free breathing	Free breathing
CMR acquisition type	2D	3D	3D	3D
Acquired/reconstructed slice thickness (mm)	1.5/1.5	1.3/0.65	1.8/0.9	1.8/0.9
Slice gap in each breath-hold acquisition	200%	n/a	n/a	n/a
Slice oversampling (%)	n/a	50	0	0
Slabs	n/a	9–11 (1 per BH)	1	1
Slices per slab	n/a	24	208	208
Partial Fourier (slice)	n/a	7/8	6/8	6/8
ipat factor	n/a	n/a	n/a	3
Shots per slice	1	1	2	2
Excitation	Slice selective	Slab selective	Non-selective	Non-selective
TR (ms)	3.3	3.4	2.4	2.4
TE (ms)	1.67	1.69	1.2	1.2
Flip angle (degrees)	95	95	90	90
Matrix	160	160	176	224
Field of view (mm)	320	350	320	400
Reconstructed in-plane spatial resolution	1.0	1.1	0.9	0.9
Bandwidth (Hz/pixel)	1359	1302	1420	1395

*n/a* not applicable; *CMR* cardiovascular magnetic resonance; *TE* echo time; *TR* repetition time

#### CTA technique

All CTA studies were performed using a dual source CT scanner (SOMATOM Definition FLASH or FORCE, Siemens Healthineers) with a gantry rotation ≤ 280 ms. The tube voltage was selected according to the patient’s body mass index and ranged between 100 and 140 kV and the tube current was automatically modulated. Typical acquisition parameters included: spiral pitch factor of 3.4; 100 ml of Iodinated contrast given at a rate of 5.5 ml/s followed by 45 ml of saline flush; prospective ECG triggered scanning performed during a single BH; reconstructed slice thickness of 0.6 mm.

### Non-rigid image registration

BH-2D and BH-SOS MRI sequences require multiple breath-holds to encompass the left atrium and PVs. Variations in inspiratory effort will often result in misregistration of images acquired in different BHs, causing artifacts in orthogonal thin maximum intensity projection reconstructions used for image interpretation. Consequently, non-rigid Elastix-based (https://elastix.isi.uu.nl/) image registration algorithms were applied to the breath-hold data sets prior to image evaluation. Given the substantial differences in acquisition technique, different image registration algorithms were used for BH 2D and 3D data.

For each 2D BH acquisition, there is a gap between adjacent slices which is filled in over successive BHs to generate a gapless volumetric data set, which is processed in Elastix as follows. We call “slice set 1” all the slices acquired during BH number 1, and so on for each BH. The entire registration process consists of repeated 3D non-rigid multi-resolution Elastix registrations (number of resolutions: 5, B-Spline transform of order 3, metric: advanced Mattes mutual information, optimizer: adaptative stochastic gradient descent, maximum number of iterations: 500, image sampler: random). In the first step, image data in slice set 1 are interpolated to the locations of slice set 2. Slice set 2 is then elastically registered to slice set 1 interpolated to the position of slice set 2. In a second step, image data from slice set 1 and slice set 2 (registered to slice set 1) are then interpolated to the locations of slice set 3. Slice set 3 is then elastically registered to this data. This process is repeated until all 5 slice sets are registered. Because sequentially acquired breath-hold 2D slice groups were overlapped, the Elastix algorithm was able to correct for through-plane as well as cranio-caudal motion.

By comparison, 3D SOS acquisitions are inherently gapless. Therefore, successive BH acquisitions are overlapped by several slices. For Elastix image registration of BH SOS data, normalized cross-correlation analysis of overlapping slices is done to identify the slices from adjacent slabs that best match. These slices are registered, and the non-rigid registration transformation is applied to the other slices in each slab.

Note that the image processing algorithms for the 2D and SOS data were totally automated with no need for user input. The combined image processing time for both the BH 2D and breath-hold 3D SOS acquisition was < 30 min.

### Image analysis

All CMR and CTA images were transferred to post-processing software (CVi42, Circle Cardiovascular Imaging, Calgary, Canada) for 3D multiplanar reconstructions (MPRs) and maximum intensity projections (MIPs). The number of PVs and the presence of variants such as common pulmonary trunk or supernumerary veins was noted.

#### Quantitative analysis

Two radiologists performed double oblique MPRs and measured the maximum and minimum cross-sectional diameters and the cross-sectional area of each of the PV ostia at the veno-atrial junction on all CMR sequences and on CTA.

Contrast-to-noise (CNR) was obtained from each of the CMR sequences using the formula 0.655 × (Sa − Sb)/noise, where Sa is the mean signal from the largest PV ostium, Sb is the background signal from the myocardium, noise is the standard deviation of air signal, and the 0.655 multiplier accounts for the Rician distribution of noise [10].

#### Qualitative analysis

CMRA image quality was assessed in 6-mm thick × 3-mm overlap axial and coronal MIPs. For healthy subjects, the free-breathing Cartesian 3D and SOS acquisitions were rated by two radiologist readers using four-point scales as follows: (a) *image quality:* 1 = at least one PV origin is non-diagnostic with severe image artifacts (including ghosts or streaking), 2 = fair visualization of all PV origins with moderate image artifacts, 3 = moderately good visualization of all PV origins with mild image artifacts, and 4 = excellent visualization of all PV origins with no significant image artifacts; (b) *pulmonary artery and vein branch vessel sharpness:* 1 = branches appear indistinct with severe blurring, 2 = branches are seen with moderate blurring, 3 = branches are seen with mild blurring, and 4 = branches appear sharp without significant blurring; and (c) *off-resonance artifacts:* 1 = off-resonance artifacts are severe precluding evaluation of at least one PV origin, 2 = off-resonance artifacts are moderate and do not preclude evaluation of any of the PV origins, 3 = off-resonance artifacts are mild and do not preclude evaluation of any of the PV origins, 4 = off-resonance artifacts are negligible and do not preclude evaluation of any of the PV origins. Ratings were done using both axial and coronal reformatted images.

For patient studies, the image quality and vessel conspicuity of the PV ostia was subjectively rated independently by two radiologists according to a 5-point scale: (1) vascular anatomy not assessable due to severe image artifacts and/or poor vascular sharpness, inadequate for diagnosis; (2) vascular anatomy assessable despite moderate image artifacts and/or moderately decreased vascular sharpness, marginally accepted for diagnosis; (3) fair image quality with mild image artifacts and/or mildly decreased vascular sharpness, acceptable for confident diagnosis; (4) good image quality with minor image artifacts and/or minimally decreased vascular sharpness, adequate for confident diagnosis; (5) excellent image quality without artifacts and with sharp vascular margins, highly confident diagnosis. When present, small accessory PV were not scored. The presence of off-resonance artifacts, which manifest as localized dark or bright signal obscuring the PV ostium, was independently rated by two radiologists according to a binary scale: (0) no significant artifacts or (1) significant artifacts.

### Statistical analysis

Agreement of CMR-measured PV maximal and minimal diameters and PV areas with respect to CTA was analyzed using the intraclass correlation coefficient (ICC) and through Bland–Altman analysis of mean differences and 95% limits of agreement. Inter-reader agreement on PV diameters and areas was analyzed using ICC. According to Koo et al. [11], ICC is interpreted as follows: < 0.5: poor; 0.5–0.75: moderate; 0.75–0.9: good; > 0.9: excellent. Friedman test and Wilcoxon signed-rank tests were used to identify differences in CNR between the different CMR sequences. Friedman tests were used to identify differences in image quality/vessel conspicuity scoring across the three CMR protocols. Inter-reader agreement for image quality/vessel conspicuity between the 2 readers was analyzed using quadratic weighted kappa test (κ) and percentage of agreement. Kappa interpretation was based on Altman guidelines [12]: κ < 0.2: poor agreement; κ = 0.21–0.40: fair agreement; κ = 0.41–0.60: moderate agreement; κ = 0.61–0.80: good agreement; κ = 0.81–1: very good agreement. A 3-sample test for equality of proportions without continuity correction was used to interrogate differences in the rates of off-resonance artifacts among the three sequences. Weighted kappa was computed in R software (version 3.3.2, R Foundation for Statistical Computing, Vienna, Austria) whereas SPSS (version 22.0. Statistical Package for the Social Sciences, International Business Machines, Inc., Armonk, New York, USA) was used for the remaining analyses. P < 0.05 was considered statistically significant.

## Results

**Fig. 1:**
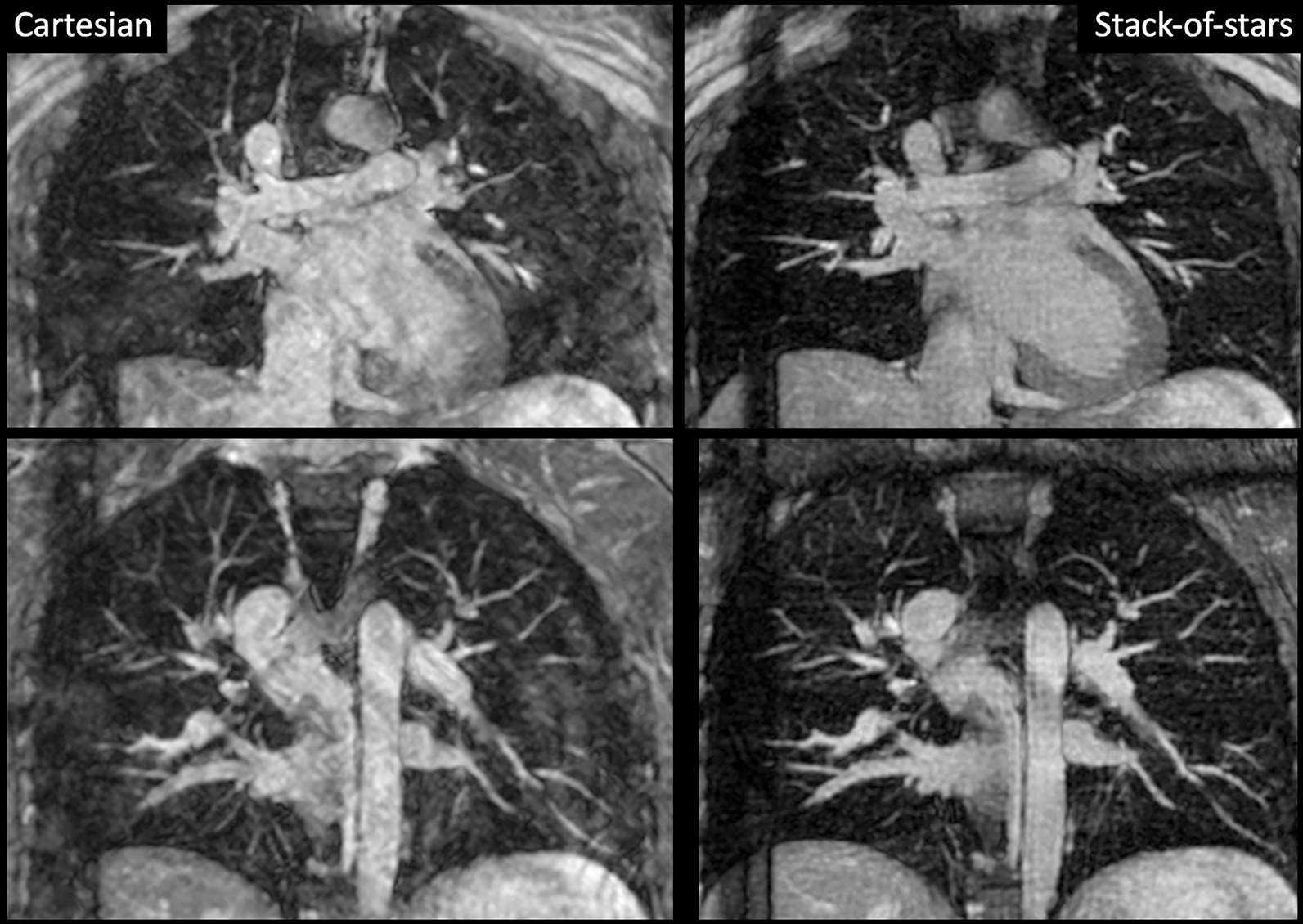
Comparison of 6-mm thick maximum intensity projection (MIPs) from coronal navigator-gated free-breathing 3D balanced steady state free precession (bSSFP) using a spatially non-selective radiofrequency (RF) excitation with Cartesian sampling (left) and radial stack-of-stars (SOS) sampling (right). Similar echo train length, spatial resolution and scan time were used for the two acquisitions; an ipat factor of 3 was used for the Cartesian acquisition. With radial imaging, ghost artifacts within the cardiac chambers and pulmonary veins are eliminated, while vessel and diaphragm sharpness are greatly improved

For FB acquisitions in the six healthy subjects using a non-selective RF excitation, FB-NS-SOS consistently outperformed Cartesian 3D with significant improvements in image quality and vessel sharpness for both readers when using both axial and coronal MIPs. An example is shown in Fig. 1. Scan time for each acquisition was on the order of 8 to 17 min. For axial MIPs, image quality scores in the form 3D FB-NS-SOS vs. 3D FB Cartesian were: 3.3 ± 0.5 vs. 2.0 ± 0.9 (reader 1, P = 0.023); 3.8 ± 0.4 vs. 2.5 ± 1.2 (reader 2, P = 0.039). For coronal MIPs, image quality scores in the form 3D FB-NS-SOS vs. 3D FB Cartesian were: 3.7 ± 0.5 vs. 2.2 ± 0.8 (reader 1, P = 0.023); 3.7 ± 0.5 vs. 2.0 ± 0.9 (reader 2, P = 0.023). For axial images, sharpness scores in the form 3D FB-NS-SOS vs. 3D FB Cartesian were: 3.2 ± 0.4 vs. 2.2 ± 0.8 (reader 1, P = 0.034); 3.7 ± 0.5 vs. 2.5 ± 0.6 (reader 2, P = 0.038). For coronal MIPs, sharpness scores in the form 3D FB-NS-SOS vs. 3D FB Cartesian were: 3.7 ± 0.5 vs. 2.3 ± 0.8 (reader 1, P = 0.023); 3.3 ± 0.5 vs. 2.5 ± 0.6 (reader 2, P = 0.025). For axial MIPs, off-resonance artifact scores in the form 3D FB-NS-SOS vs. 3D FB Cartesian were: 3.7 ± 0.8 vs. 3.5 ± 0.8 (reader 1, P = 0.317); 3.8 ± 0.4 vs. 3.7 ± 0.8 (reader 2, P = 0.317). For coronal MIPs, off-resonance artifact scores in the form 3D FB-NS-SOS vs. 3D FB Cartesian were: 3.7 ± 0.8 vs. 3.5 ± 0.8 (reader 1, P = 0.317); 3.8 ± 0.4 vs. 3.5 ± 0.8 for 3D Cartesian (reader 2, P = 0.157). No significant differences in off-resonance artifacts were observed between the FB-NS-SOS and Cartesian 3D protocols.

**Fig. 2:**
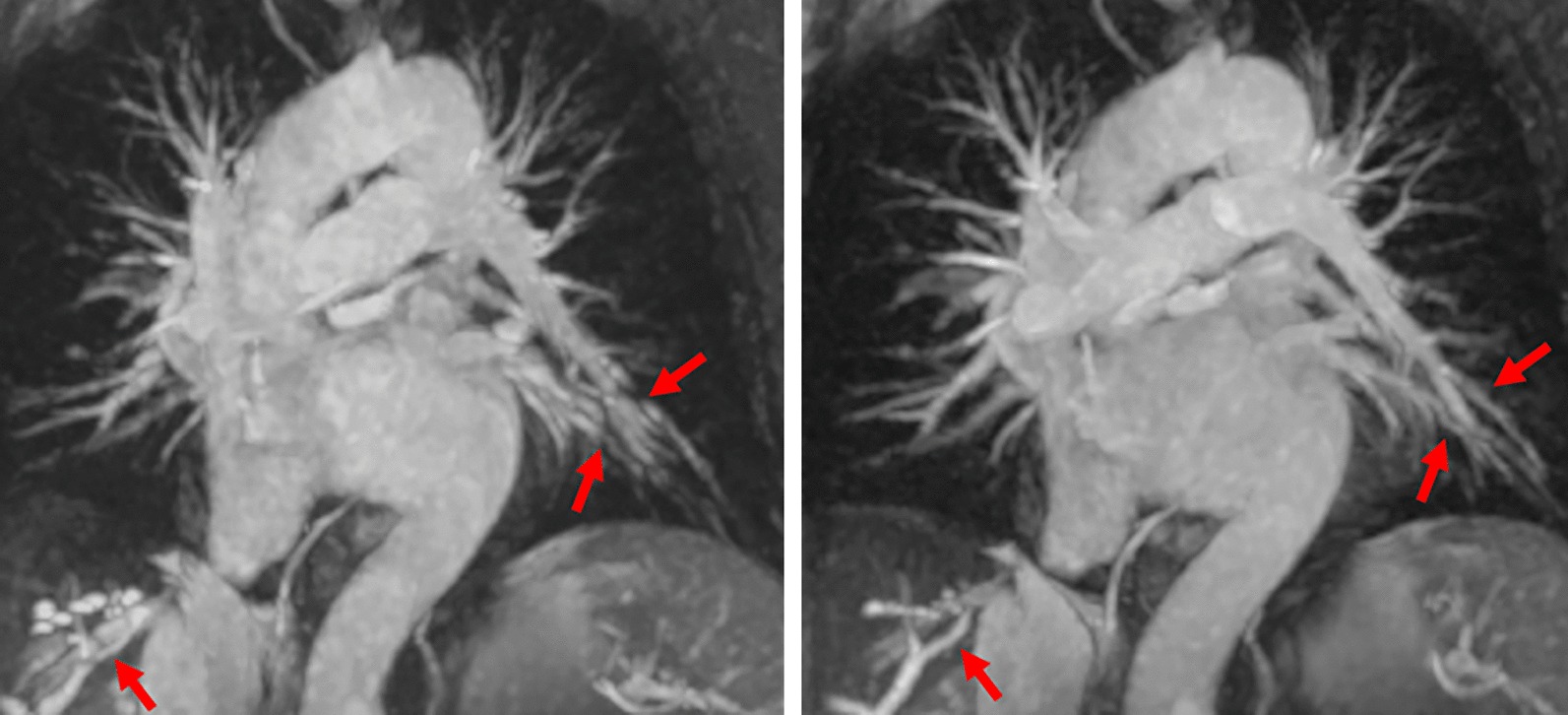
Left: Full-thickness maximum intensity projection of overlapping thin-slab BH-SOS acquired in 9 separate breath-holds shows misregistration artifacts (arrows) due to varying inspiratory depth across the breath-holds. Right: after Elastix-based image registration, there is substantially better alignment of the vessels from the multiple slabs, resulting in improved maximum intensity projection image quality

For BH acquisitions, the effectiveness of the non-rigid Elastix-based image registration is illustrated in Fig. 2. Elastix-based registration corrected misalignment of adjacent slices and slabs due to small differences in breath-hold positions.

From a total of 27 patients, 15 had conventional PV anatomy, 10 had a left common PV trunk and 2 had a distinct right middle PV. Table 2 summarizes the ICC values for the maximal and minimal diameters and for the areas of each vessel and of all vessels grouped together on each of BH-2D, BH-SOS and FB-NS-SOS compared to CTA. For PV maximal diameter, there was overall good agreement between BH-2D and CTA (ICC = 0.87 for reader 1/ICC = 0.83 for reader 2), excellent agreement between BH-SOS and CTA (ICC = 0.90/0.91), and good to excellent agreement between FB-NS-SOS and CTA (ICC = 0.87/0.94), for all vessels grouped together. For PV minimal diameter, there was overall moderate agreement between BH-2D and CTA (ICC = 0.63/0.74), good agreement between BH-SOS and CTA (ICC = 0.76/0.78) and good to excellent agreement between FB-NS-SOS and CTA (ICC = 0.84/0.92), all vessels grouped together. For PV area, there was overall good agreement between BH-2D and CTA (ICC = 0.79/0.83), good to excellent agreement between BH-SOS and CTA (ICC = 0.88/0.91) and excellent agreement between FB-NS-SOS and CTA (ICC = 0.90/0.95). Except for PV maximal diameter for reader 1, FB-NS-SOS provided the largest ICC values. Table 3 summarizes the ICC values between the two readers for the maximal and minimal diameters and for the areas of each vessel and of all vessels grouped together on each non-contrast CMRA sequence. FB-NS-SOS provided the best inter-rater agreement. Table 4 summarizes the mean difference and limits of agreement between each non-contrast CMRA compared to CTA obtained with Bland–Altman analysis. With respect to CTA, BH-SOS provided the smallest mean difference for maximal diameter and area, where FB-NS-SOS provided the smallest mean difference for minimal diameter.

**Table 2:** ICC of minimum (D_min_) and maximum (D_max_) PV diameters and areas measured during diastole on each non-contrast CMRA sequence compared to CTA

		BH-2D		BH-SOS		FB-NS-SOS	
		Reader 1	Reader 2	Reader 1	Reader 2	Reader 1	Reader 2
Right superior	D_max_	0.88^c^	0.79^c^	0.89^c^	0.75^c^	0.94^c^	0.93^c^
	D_min_	0.54^b^	0.81^c^	0.63^b^	0.52^b^	0.79^c^	0.92^c^
	Area	0.79^b^	0.80^c^	0.78^a^	0.80^c^	0.89^b^	0.92^c^
Right inferior	D_max_	0.52^c^	0.56^c^	0.59^c^	0.74^c^	0.62^b^	0.83^c^
	D_min_	0.43^b^	0.58^c^	0.64^c^	0.64^b^	0.74^b^	0.87^c^
	Area	0.60^c^	0.71^c^	0.77^c^	0.86^c^	0.83^c^	0.91^c^
Left superior	D_max_	0.76^c^	0.66^c^	0.82^c^	0.93^c^	0.92^c^	0.95^c^
	D_min_	0.72^c^	0.77^c^	0.88^c^	0.96^c^	0.93^c^	0.98^c^
	Area	0.69^c^	0.76^c^	0.87^c^	0.94^c^	0.93^c^	0.96^c^
Left inferior	D_max_	0.88^c^	0.78^c^	0.83^c^	0.85^c^	0.91^c^	0.93^c^
	D_min_	0.70^c^	0.77^c^	0.76^c^	0.89^c^	0.87^c^	0.87^c^
	Area	0.81^c^	0.81^c^	0.87^c^	0.89^c^	0.88^b^	0.94^c^
Left common	D_max_	0.89^c^	0.83^b^	0.93^c^	0.91^b^	0.87^a^	0.83^a^
	D_min_	0.69^a^	0.75^b^	0.66^a^	0.61^a^	0.94^a^	0.83^a^
	Area	0.80^b^	0.87^c^	0.87^a^	0.86^b^	0.09	0.23
All PVs	D_max_	0.87^c^	0.83^c^	**0.90**^**c**^	0.91^c^	0.87^c^	**0.94**^**c**^
	D_min_	0.63^c^	0.74^c^	0.76^c^	0.78^c^	**0.84**^**c**^	**0.92**^**c**^
	Area	0.79^c^	0.83^c^	0.88^c^	0.91^c^	**0.90**^**c**^	**0.95**^**c**^

All images were acquired in a coronal plane. Largest ICC values for all pulmonary veins and for each reader are shown in bold

^a^P < 0.05; ^b^P < 0.01; ^c^P < 0.001; *BH* breath hold; *FB* free breathing, *SOS* stack of stars

**Table 3:** ICC of PV diameters and areas on each non-contrast CMRA sequence between the two readers

		BH-2D	BH-SOS	FB-NS-SOS
Right superior	D_max_	0.84^c^	0.92^c^	0.96^c^
	D_min_	0.90^c^	0.85^c^	0.93^c^
	Area	0.92^c^	0.95^c^	0.98^c^
Right inferior	D_max_	0.83^c^	0.82^c^	0.95^c^
	D_min_	0.76^c^	0.67^b^	0.94^c^
	Area	0.85^c^	0.94^c^	0.99^c^
Left superior	D_max_	0.39	0.84^c^	0.96^c^
	D_min_	0.56^b^	0.77^c^	0.98^c^
	Area	0.94^c^	0.67^b^	0.99^c^
Left inferior	D_max_	0.85^c^	0.95^c^	0.96^c^
	D_min_	0.77^c^	0.94^c^	0.94^c^
	Area	0.94^c^	0.99^c^	0.99^c^
Left common	D_max_	0.92^c^	0.98^c^	0.89^a^
	D_min_	0.92^c^	0.89^b^	0.96^b^
	Area	0.93^c^	0.91^b^	0.28
All PVs	D_max_	0.88^c^	0.95^c^	**0.97**^**c**^
	D_min_	0.81^c^	0.86^c^	**0.95**^**c**^
	Area	0.93^c^	0.95^c^	**0.99**^**c**^

Largest ICC values for all pulmonary veins are shown in bold

^a^P < 0.05; ^b^P < 0.01; ^c^P < 0.001

**Table 4:** Bland–Altman analysis of all PV ostial diameters and areas on each non-contrast CMRA sequence compared to CTA for both readers

		BH-2D	BH-SOS	FB-NS-SOS	CT mean value
D_max_ (mm)	Reader 1	0.81 (− 4.52, 6.14)	**0.55** (− 3.93, 5.04)	0.95 (− 3.88, 5.78)	23.22
	Reader 2	0.74 (− 5.10, 6.60)	**0.23** (− 4.01, 4.47)	0.58 (− 2.87, 4.03)	23.37
D_min_ (mm)	Reader 1	0.47 (− 5.49, 6.44)	0.33 (− 4.30, 4.97)	− **0.13** (− 3.87, 3.62)	17.06
	Reader 2	0.89 (− 4.91, 6.68)	0.35 (− 4.22, 4.92)	**0.10** (− 2.65, 2.85)	17.34
Area (mm^2^)	Reader 1	20.69 (− 119.77, 161.16)	**6.95** (− 94.58, 108.48)	13.10 (− 82.88, 109.09)	303.93
	Reader 2	25.07 (− 99.89, 150.04)	**8.50** (− 81.04, 98.04)	8.83 (− 64.02, 81.69)	313.67

Data are presented as mean difference (95% limits of agreement). The smallest mean differences with respect to CTA are shown in bold. The mean values for PV ostial diameters and area by CTA are provided in the right-most column

Friedman test showed significant differences in CNR across the non-contrast CMRA sequences (P = 0.002). Wilcoxon signed-rank test demonstrated significantly higher CNR with BH-SOS (mean = 11.04) compared to BH-2D (mean = 6.02; P = 0.007) and FB-NS-SOS (mean = 5.29; P = 0.002). There was no significant difference in CNR between BH-2D and FB-NS-SOS (P = 0.345).

**Fig. 3:**
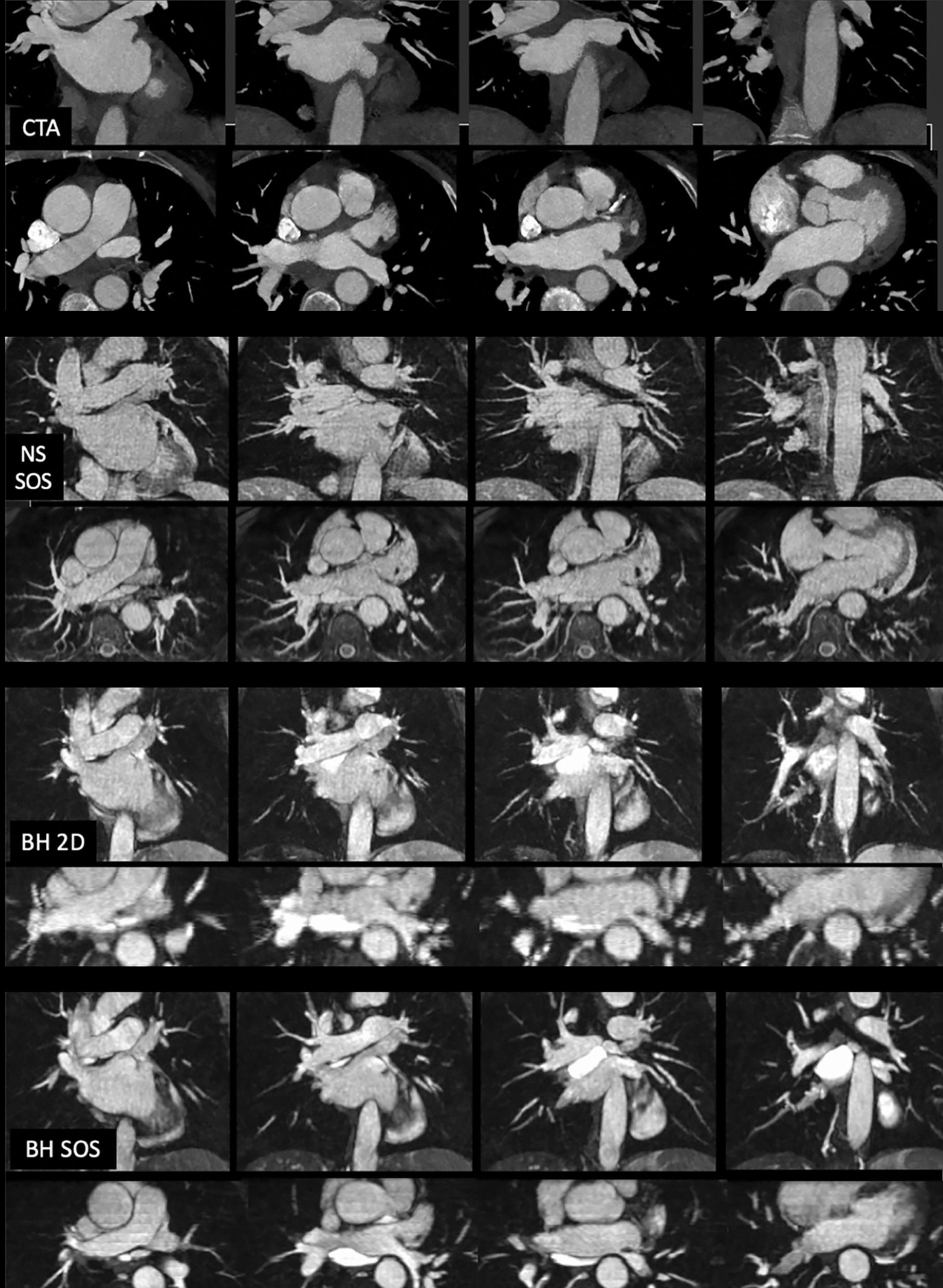
Comparison of maximum intensity projections from coronal and axial computed tomography angiography (CTA), navigator-gated free-breathing 3D stack-of-star bSSFP using a spatially non-selective RF excitation (NS-SOS), breath-hold 2D radial bSSFP (BH-2D) and breath-hold 3D stack-of-stars bSSFP (BH-SOS). For NS-SOS, BH-2D and BH-SOS, both axial and coronal maximum intensity projections were reconstructed from a coronal scan. All three non-contrast CMRA techniques show good image quality in the coronal maximum intensity projections. However, more vascular blurring is apparent in the BH-2D axial maximum intensity projections due to the larger slice thickness and worse slice profile compared with the 3D acquisitions

BH-SOS and FB-NS-SOS showed better image quality than BH-2D for both readers for the mean score of image quality of the PV ostia (P < 0.05) (Table 5) (Fig. 3).

**Table 5:** Comparison of the image quality/vessel conspicuity of the PV ostia

	BH-2D	BH-SOS	FB-NS-SOS	P value
Reader 1	3.5 ± 0.7	4.1 ± 0.5	4.1 ± 0.5	0.006
Reader 2	3.4 ± 0.7	4.0 ± 0.5	4.1 ± 0.6	< 0.001

The mean score of 4 veins is shown. Image quality/vessel conspicuity was rated as: 1 = poor; 2 = marginal; 3 = fair; 4 = good; 5 = excellent. Data presented as mean ± standard deviation

**Fig. 4:**
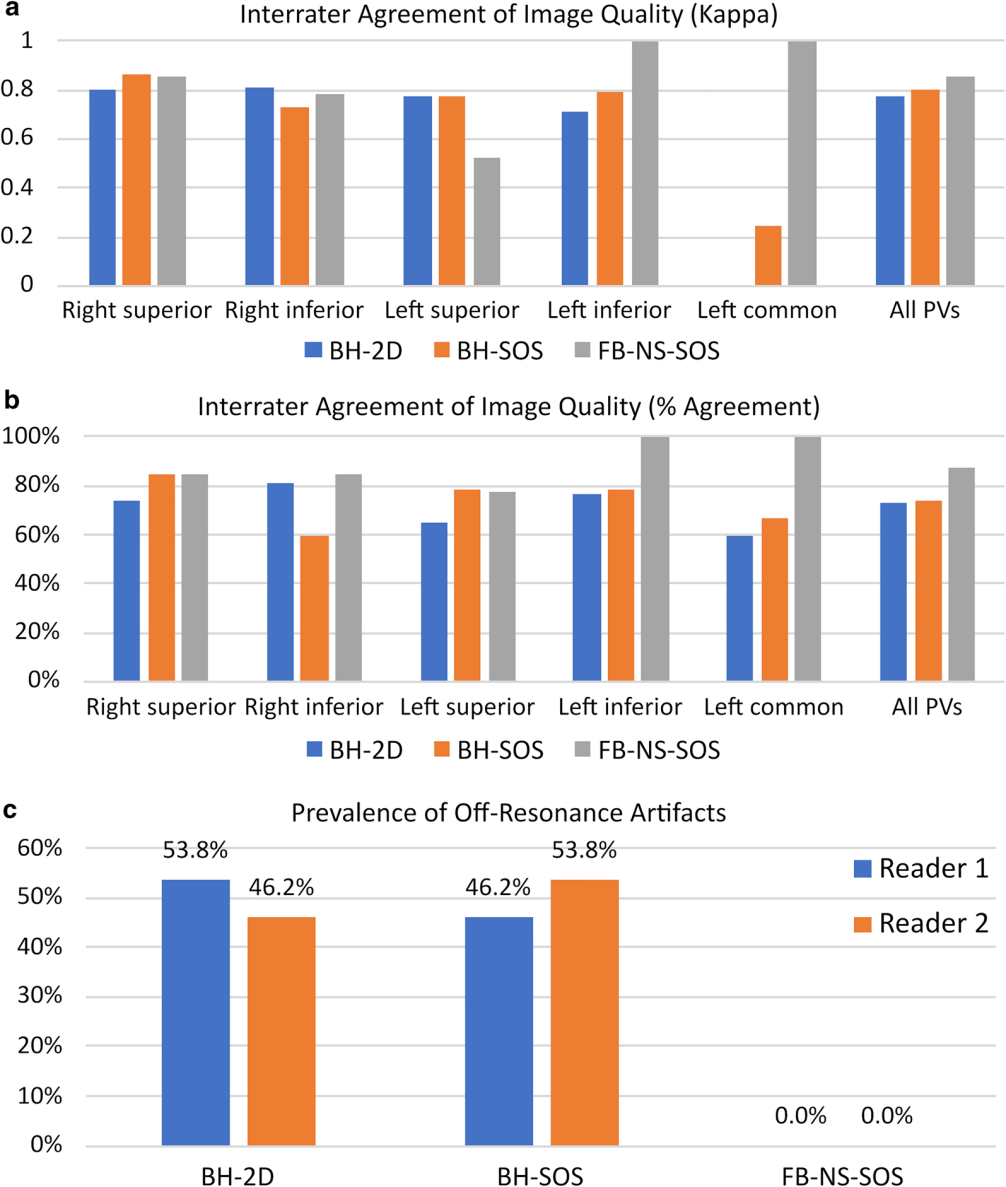
Bar plots showing interrater agreement as assessed by **a** quadratic weighted kappa and **b** percentage agreement, and **c** prevalence of off-resonance artifacts at the level of the right inferior pulmonary vein

The inter-rater agreement for image quality/vessel conspicuity scores was good to very good using either BH-2D, BH-SOS or FB-NS-SOS for all the vessels except for the left common trunk on both BH-2D and BH-SOS and for the left superior PV on FB-NS-SOS (Fig. 4a, b).

**Fig. 5:**
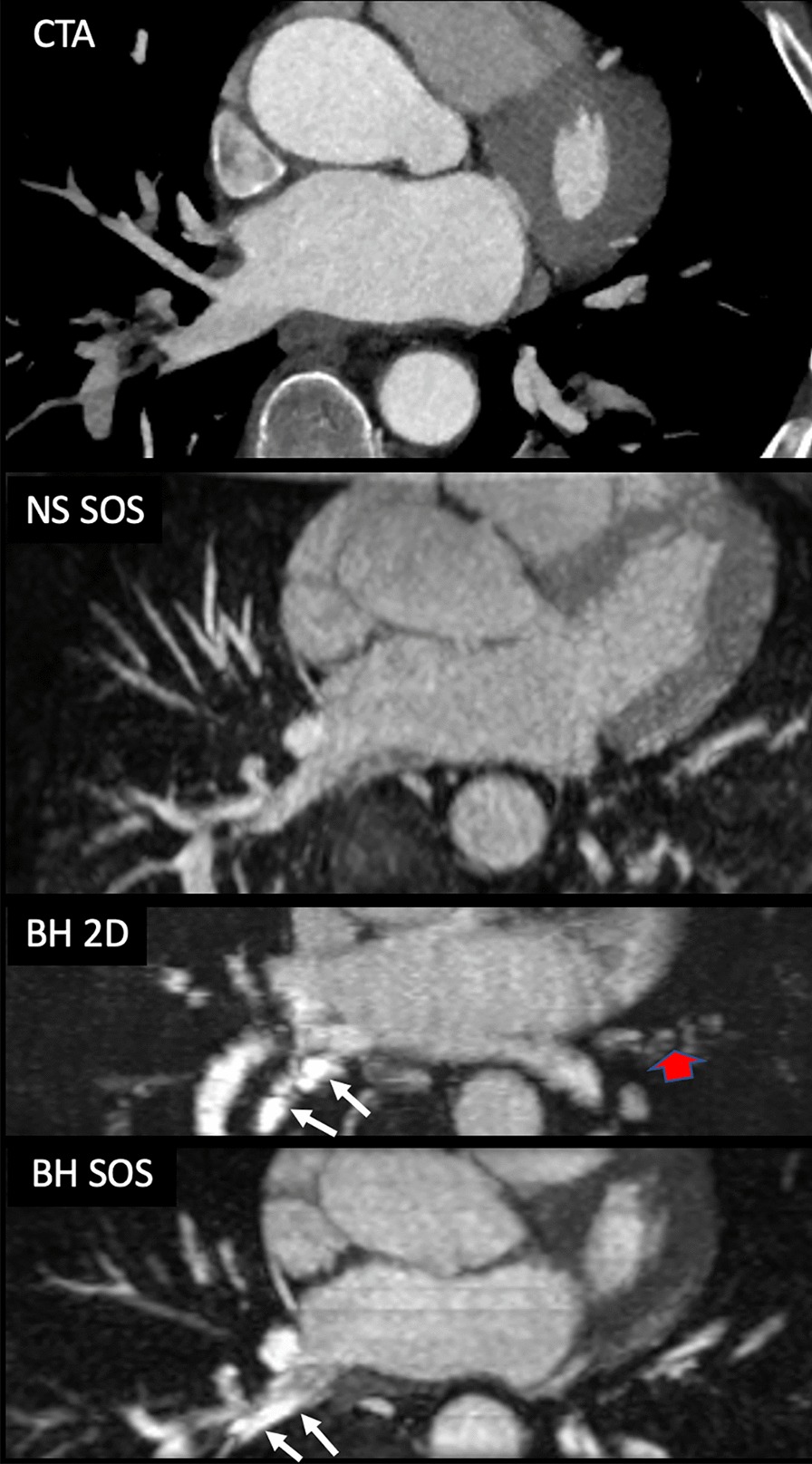
Example of a subject undergoing evaluation prior to radiofrequency catheter ablation with CTA and non-contrast CMRA. Axial maximum intensity projections are shown, reconstructed from the coronal acquisitions. Note the off-resonance artifact seen at the level of the right inferior pulmonary vein ostium on both BH-2D and BH-SOS, which degrades the image quality and sharpness of the vessel contour. There is also some residual misregistration artifact with BH-2D (wide red arrow). However, the ostial diameter can still be adequately measured. NS-SOS shows sharp margins of the right inferior pulmonary vein ostium with negligible off-resonance artifact

Off-resonance artifacts were mainly observed at the level of the right inferior PV on BH-2D and BH-SOS for both readers. There was a statistically significant difference in the proportion of subjects with off-resonance artifacts at the right inferior PV between the three different non-contrast CMRA sequences (P = 0.007 for reader 1 and P = 0.007 for reader 2), with no substantial off-resonance artifacts detected using FB-NS-SOS among the 13 subjects that underwent the three non-contrast CMRA sequences (Figs. 4c, 5).

Rarely, off-resonance artifacts were seen at other PV ostia: one case at the left superior PV on BH-2D and BH-SOS sequences for both readers in which the left main bronchus was seen to course in close proximity to the left superior PV ostium; one case at the left inferior PV only on BH-2D for reader 1 and on BH-2D and BH-SOS for reader 2 of unclear etiology; and one case at the right superior PV on BH-SOS for reader 2 in which the lung parenchyma extends deep adjacent to the right superior PV ostium.

## Discussion

To our knowledge, radial acquisition techniques have not previously been applied to the pre-procedural evaluation of the PVs in patients with a history of AF. Moreover, only FB acquisitions have been reported for this clinical indication. In comparing a conventional FB Cartesian 3D imaging strategy for non-contrast PV imaging to one using a radial SOS k-space trajectory in healthy subjects, we found that the SOS acquisition provided a visually obvious and statistically significant improvement in the image quality and vessel sharpness. This result is consistent with previous work demonstrating that radial-based non-contrast CMRA acquisition techniques provide substantially better image quality, with improved vessel sharpness and elimination of ghost and fold over artifacts, compared with Cartesian-based techniques [13]. The reduction in flow and motion artifacts arises largely from oversampling of the center of k-space [8]. Moreover, radial techniques preserve spatial resolution despite the use of high radial undersampling factors, which allows for considerable flexibility regarding scan acceleration. Consequently, only radial k-space trajectories were used to image patients in this study.

In order to help determine a preferred imaging strategy to depict the PVs, we compared the diagnostic performance of several radial-based acquisition techniques including both BH and FB imaging strategies, using CTA as the reference standard. This study showed that both BH and FB radial CMRA were generally able to depict PV anatomy and provide diagnostic image quality, with best vessel sharpness obtained with 3D radial acquisition technique (BH-SOS and FB-NS-SOS). The three studied sequences allowed accurate diameter measurements that were in at least good agreement with CTA.

CTA and CE-CMRA are both well-established imaging modalities for depicting the PVs [14-17]. Non-contrast CMRA using a bSSFP readout provides a potentially useful alternative for patients in whom contrast agents are relatively contraindicated. However, bSSFP is sensitive to static magnetic field inhomogeneities at the interface between the lungs and PV ostia [18, 19]. Consequently, prior non-contrast studies have relied primarily on the use of a Cartesian 3D bSSFP acquisition incorporating a spatially non-selective RF excitation. The use of a short-duration non-selective RF excitation decreases the repetition time (e.g. by about 33% with our sequence implementation), which in turn reduces the sensitivity of the bSSFP readout to off-resonance artifacts [6, 7]. However, there are some limitations to these prior studies. For instance, Krishnam et al. [7] used relatively thick slices (e.g. 3-mm), which compares unfavorably to the submillimeter slice thicknesses afforded by CTA. Moreover, image evaluation was limited in that the PV ostia were measured only in the supero-inferior direction from the coronal source images, which is not the current practice with CTA. François et al. [6] evaluated a similar free-breathing acquisition technique in 20 patients and reported good image quality and accurate measurements of PV diameters. By comparison, the present study used much thinner slices. The reconstructed slice thicknesses of 0.65 mm with BH-SOS and 0.9 mm with FB-NS-SOS are comparable to those used with CTA and several times smaller than those used in the previous reports. Moreover, prior reports generally used ungated CE-CMRA as the reference method. While CE-CMRA can provide adequate image quality, it is suboptimal as a reference standard because spatial resolution is inferior to CTA and the use of an ungated acquisition technique results in considerable blurring of cardiac structures, potentially including the PV ostia. In the present study, ECG-gated CTA with state-of-the-art dual source scanners was used as the reference standard in order to provide the best image quality and spatial resolution.

Artifacts that we attribute to off-resonance effects were primarily observed at the right inferior PV ostium on BH-2D and BH-SOS but were negligible on FB-NS-SOS. Hu et al. reported significantly more off-resonance artifacts at the right inferior PV than other PVs and they proposed that differences in lung morphology and distance between the PV and air-containing lung tissue were potential etiologies for the artifacts [20]. Shigenaga et al. reported that artifacts were worse with 3D vs. 2D bSSFP and they considered this related to greater inflow refreshment with 2D [21].

Each acquisition technique has certain benefits and drawbacks. Compared with 2D radial, SOS allows for the acquisition of thinner slices with more rectangular slice profiles [22]. Unlike the FB-NS-SOS approach which uses a non-selective RF excitation that encompasses the entire imaging volume, both 2D radial and thin-slab SOS CMRA only image a relatively thin volume of tissue in each BH. The thin imaging volume permits substantial inflow-related enhancement for the PV, which enhances PV conspicuity. Moreover, the image quality of BH acquisitions is not degraded by irregular breathing patterns, although off-resonance artifacts remain a limitation for both 2D and 3D BH acquisitions.

On the other hand, FB acquisitions benefit from the combination of a shorter TR along with the ability to acquire two shots, resulting in an acquisition window that is substantially shorter than for the BH acquisitions. The shorter acquisition window has the potential advantage of reducing blurring from cardiac motion. Another drawback of BH compared with FB is the need to use retrospective non-rigid registration algorithms to compensate for variations in inspiratory depth among the multiple BH.

FB acquisitions using a spatially non-selective RF excitation do not benefit from inflow of unsaturated spins into the PV, since the large flip angle RF pulse saturates all spins within the excitation volume of the body coil. Given that the typical RR interval is much shorter than the T1 relaxation time of blood (~ 1500 ms at 1.5 T [23]), triggering to every R-wave results in substantial spin saturation of the PV. Triggering to every second R-wave allows more time for T1 relaxation and thereby increases PV signal and contrast with non-vascular background tissues. However, the trade-off is a doubling of scan time. Scan time could be reduced through the use of parallel imaging along the slice direction and/or compressed sensing [24]. Finally, it should be noted that relative advantages of time-resolved CE-CMRA versus non-contrast CMRA are shorter scan time, suppression of fluid signal in the pericardial recesses, as well as selective enhancement of the left atrium and PV which simplifies the process of segmentation of these structures.

There are several limitations of our study. First, while the imaging parameters for the FB Cartesian and SOS bSSFP acquisitions were matched as far as was practical, they were likely not optimal with regard to readout duration or with regard to use of advanced techniques such as variable density k-space sampling and compressed sensing. Second, whereas a T2 preparation has been used in prior studies, we chose not to use this technique because of our preliminary observation that it tended to exacerbate off-resonance artifacts near the PV ostia. Third, the sample size is relatively small and not every subject underwent all three non-contrast CMRA techniques. Finally, only the anatomy, visibility and measurements of the PV ostia were evaluated while other parameters (such as left atrial appendage morphology, presence of atrial thrombus, relationship to esophageal wall) that can be relevant prior to a PV isolation procedure were not formally assessed.

## Conclusion

While further study in a larger patient population is needed, our results suggest that a FB SOS bSSFP technique is advantageous in providing accurate depiction of PV anatomy and ostial measurements without significant degradation from off-resonance artifacts. Moreover, image quality and vessel sharpness using a SOS k-space trajectory are superior to a Cartesian 3D trajectory. For patients in whom respiratory gating is unsuccessful due to irregular breathing patterns or other factors, a BH thin-slab SOS technique with retrospective motion correction appears may be a useful alternative.

## Data Availability

Not applicable.

## Abbreviations

AF: Atrial fibrillation
BH: Breath hold
bSSFP: Balanced steady-state free precession
CE: Contrast enhanced
CMRA: Cardiovascular magnetic resonance angiography
CTA: Computed tomography angiography
CNR: Contrast-to-noise ratio
ECG: Electrocardiogram
FB: Free breathing
GBCA: Gadolinium based contrast agent
ICC: Intraclass correlation
MIP: Maximum intensity projection
MPR: Multiplanar reconstruction
PV: Pulmonary vein
SOS: Stack-of-stars
